# Efficacy of mindfulness-based interventions for children and adolescents with attention deficit hyperactivity disorder: a systematic review and meta-analysis

**DOI:** 10.3389/fpsyt.2026.1894822

**Published:** 2026-09-16

**Authors:** Fang Shen, Hui Zhou, Tongming Zhang

**Affiliations:** Department of Pediatric Rehabilitation, Key Laboratory of Birth Defects and Related Diseases of Women and Children, Ministry of Education (MOE), West China Second University Hospital, Sichuan University, Chengdu, Sichuan, China

**Keywords:** attention deficit hyperactivity disorder, children and adolescents, meta-analysis, mindfulness-based interventions, systematic review

## Abstract

**Background and objective:**

Attention deficit hyperactivity disorder (ADHD) is a common neurodevelopmental disorder in childhood. Although mindfulness-based interventions (MBIs) have been proposed as promising non-pharmacological treatments, evidence regarding their efficacy in children and adolescents with ADHD remains inconsistent.

**Methods:**

A systematic search of PubMed, Embase, the Cochrane Library, Scopus, and Web of Science was conducted up to December 31, 2025, supplemented by manual screening of reference lists. Eligible studies were randomized controlled trials (RCTs) in which mindfulness-based intervention was the primary treatment for children or adolescents with ADHD, even alongside other therapies. Study selection followed the Preferred Reporting Items for Systematic Reviews and Meta-Analyses (PRISMA) guidelines. Risk of bias was assessed using the Cochrane RoB 1.0 tool. Meta-analyses employed random-effects or fixed-effects models based on heterogeneity.

**Results:**

Fourteen randomized controlled trials were included in the meta-analysis. The results demonstrated that MBIs significantly alleviated core ADHD symptoms in children (standardized mean difference (SMD) = -1.30; 95% confidence interval (95% CI) [-2.14, -0.45]; p = 0.003). However, the heterogeneity was considerably high (*I²* = 91%), and publication bias could not be excluded (as suggested by funnel plot/Egger’s test, if available). Regarding neurocognitive functions, MBIs showed potential benefits in improving attention network function (SMD = 0.44; 95% CI [0.01, 0.87]; p = 0.04; *I²* = 88%) and executive function (SMD = 0.45; 95% CI [0.02, 0.88]; p = 0.04; *I²* = 84%), though the high heterogeneity (*I²* > 75%) and possible publication bias warrant cautious interpretation. No significant effects were observed on emotional problems, social problems, externalizing behavioral problems (including oppositional defiant, disruptive, and aggressive behaviors), or mindfulness skills.

**Conclusions:**

This systematic review suggests that MBIs may improve core ADHD symptoms and neurocognitive functions (including attention and executive functions) in children and adolescents. Yet, effects on emotional health, social relationships, externalizing behaviors, and mindfulness skills remain inconclusive. While suggesting that MBIs hold promise as a potential adjunctive intervention alongside standard treatments, significant heterogeneity underscores the need for further high-quality research to confirm these therapeutic benefits.

**Systematic review registration:**

https://www.crd.york.ac.uk/PROSPERO/view/CRD420251171407, identifier CRD420251171407.

## 1 Introduction

Attention deficit hyperactivity disorder (ADHD) is a common, highly heritable neurodevelopmental disorder in childhood (1–5). ADHD is characterized by a persistent and pervasive pattern of inattention, hyperactivity/impulsivity, or both that interferes with functioning or development (6). It affects patients’ academic, occupational, emotional, and social skills, imposing a substantial burden on families and society (7–9). The estimated global prevalence among children and adolescents is approximately 7.2% (10, 11). Consequently, this condition has emerged as a major global public health concern, and strategies for the effective treatment of ADHD have become a subject of widespread attention.

Currently, first-line pharmacotherapies for ADHD include stimulant medications, specifically methylphenidate (MPH) (12, 13). However, up to one-third of children with ADHD do not derive sufficient benefit from stimulants, and their use is frequently accompanied by side effects such as loss of appetite, sleep disorders, and slowed growth, thereby resulting in low medication adherence rates among children and adolescents with ADHD (14–19). Consequently, current clinical guidelines for ADHD recommend personalized multimodal treatment approaches, particularly for children. To expand therapeutic options for children with ADHD, several non-pharmacological interventions have been developed.

An increasing number of studies highlight the efficacy of psychotherapy in ameliorating ADHD symptoms (20, 21), and mindfulness interventions are increasingly being regarded as a promising treatment approach (22). Although several systematic reviews and meta-analyses regarding the effects of mindfulness-based interventions (MBIs) on patients with ADHD have been published, yielding some promising yet inconclusive results. Most systematic reviews and meta-analyses suggest that MBIs confer potential benefits on ADHD symptoms in pediatric populations (23–28). However, the results of a systematic review indicated that due to the low methodological quality of the included studies, no definitive conclusions could be drawn regarding the efficacy of meditation-based mind-body interventions for children with ADHD (29). Evidence concerning the efficacy of mindfulness interventions on executive function, behavioral issues, anxiety, depressive symptoms, and mindfulness-related constructs in pediatric ADHD remains equivocal (24, 26, 28). Differences in study findings may be associated with baseline characteristics of study participants, features of mindfulness interventions, control group conditions, and outcome assessment tools.

Although previous systematic reviews and meta-analyses have investigated MBIs in children and adolescents with ADHD, significant research gaps remain. First, earlier reviews predominantly focused on core symptoms, with evidence regarding neurocognitive functions—specifically executive function components such as inhibitory control, working memory, and cognitive flexibility—remaining underexplored or inconclusive due to a limited number of available studies at the time. For instance, the review highlighted methodological limitations in earlier studies but could not draw definitive conclusions (29), while more recent meta-analyses primarily included literature up to August 2022 (27). Consequently, they missed a growing body of recent RCTs published between 2023 and 2025 that utilize more rigorous methodologies and standardized protocols. Second, the efficacy of MBIs on broader functional outcomes, such as emotional problems, social relationships, and mindfulness skills, has yielded inconsistent findings across previous syntheses. The heterogeneity in intervention types and control groups in earlier studies often obscured the specific benefits of mindfulness, necessitating a re-evaluation with a larger, updated sample.

To address these gaps and provide updated evidence-based data, the present meta-analysis was conducted with two main novel contributions: (1) incorporating the latest RCTs published up to December 31, 2025 to expand the evidence base and enhance statistical power; and (2) comprehensively evaluating not only core symptoms but also broader functional outcomes, including detailed subdomains of executive function and attention networks. Unlike previous reviews that often treated neurocognitive outcomes as secondary, this study places equal emphasis on these functional measures to determine whether MBIs can improve cognitive impairments in children and adolescents with ADHD.

Therefore, the primary objective of this study is to conduct an updated systematic review and meta-analysis to determine the efficacy of MBIs on core ADHD symptoms, neurocognitive functions, and psychosocial outcomes in children and adolescents. By doing so, this review aims to clarify previous inconsistencies, and provide clinicians with updated evidence to support the integration of mindfulness into multimodal treatment plans for pediatric ADHD.

## 2 Methods

### 2.1 Protocol registration

This study followed the Preferred Reporting Items for Systematic Reviews and Meta-Analyses (PRISMA) guidelines (30) and was registered in the International Prospective Register of Systematic Reviews (PROSPERO) database (CRD420251171407) (available at: https://www.crd.york.ac.uk/PROSPERO/view/CRD420251171407).

### 2.2 Search strategy

Studies were identified by searching PubMed, Embase, Cochrane Library, Scopus, and Web of Science from the inception of each database to December 31, 2025. Additionally, we performed a search in Google Scholar using keywords such as “ADHD” and “mindfulness” to capture grey literature. Finally, we manually screened the reference lists of the retrieved articles from the selected studies to identify further relevant articles. The search terms primarily defined our population of interest (i.e., children and adolescents with ADHD) and the exposures of interest (e.g., MBIs). Detailed search terms are provided in the supplementary information (see **Supporting Information S1**).

### 2.3 Eligibility criteria

The eligibility criteria were defined according to the PICOS framework as follows:

#### 2.3.1 Types of participants

We included children and adolescents aged 6 to 18 years who had been formally diagnosed with ADHD by a clinician. There were no restrictions regarding comorbid organic diseases or participant gender. Studies involving mixed populations (e.g., participants with ADHD and other comorbidities) were included only if the data for the ADHD group were reported separately.

#### 2.3.2 Types of interventions

We included studies that evaluated MBIs alone or in combination with another treatment. Major MBIs included, but were not limited to, Mindfulness-Based Stress Reduction (MBSR), Mindfulness-Based Cognitive Therapy (MBCT), mindfulness-based movement therapies (e.g., Tai Chi), and mindfulness meditation.

#### 2.3.3 Types of comparators

Comparators included no intervention, waitlist, treatment as usual (TAU), or other types of active interventions (e.g., cognitive behavioral therapy, psychological treatment).

#### 2.3.4 Types of outcomes

Outcome measures encompassed any health-related outcomes, such as ADHD symptoms, neuropsychological function, emotional problems, social problems, behavioral problems, or mindfulness skills. Studies were required to provide quantitative data from standardized psychometric scales pre- and post-intervention.

#### 2.3.5 Types of study designs

We included randomized controlled trials (RCTs).

Exclusion Criteria Studies were excluded if they were: (1) case reports, conference abstracts, reviews, or systematic reviews; (2) studies involving animal models or *in vitro* experiments; (3) published in non-English languages; (4) studies with incorrect, missing, or inaccessible data; or (5) studies involving non-mindfulness interventions or interventions lacking sufficient detail.

### 2.4 Study selection and data extraction

After removing duplicate articles, two researchers independently evaluated the search results using EndNote X9 in accordance with the inclusion and exclusion criteria to ensure the accuracy of the systematic search. First, they excluded irrelevant studies based on titles and abstracts, and then retrieved and analyzed the full texts of all studies deemed relevant. Disagreements regarding data extraction were resolved through discussion to reach a consensus; if no consensus could be reached, a third researcher was consulted to make the final decision. All data were independently extracted by the two researchers and recorded in a pre-designed standardized Excel spreadsheet. The extracted information included basic details (year of publication, first author, and country where data were collected), study type, participant characteristics (age range, mean age, gender, and sample size), intervention features (intervention type, frequency, and duration), outcome measures (ADHD symptoms, neurocognitive functions, behavioral problems, emotional problems, social functions, mindfulness skills, etc.), and the number of follow-up assessments. Corresponding authors of the included studies could be contacted via email to obtain any missing information. For missing standard deviations that could not be obtained from the authors, we calculated them from available statistical data (e.g., t-values, p-values, or 95% confidence intervals) using standard formulas provided in the Cochrane Handbook. Statistical variables including mean values, standard deviations (SD), and sample sizes were also extracted. If a study contained multiple intervention arms, we combined the relevant intervention groups to create a single pair-wise comparison to avoid double-counting the control group.

### 2.5 Quality assessment

The risk of bias was assessed using the Cochrane Collaboration tool for assessing the risk of bias embedded in Review Manager 5.4 version. The assessment of the risk of bias was performed by two independent researchers. In case of disagreement, a third researcher was consulted to reach a final decision (24).

### 2.6 Meta-analytic procedures

Meta-analysis was performed using Review Manager version 5.4 software. Outcome measures evaluated using scale scores as continuous data included the mean and standard deviation of all experiments. The standardized mean difference (SMD) was calculated by dividing the difference in means between the intervention and control groups by the pooled standard deviation. Due to differences in the rating scales, SMD was calculated to standardize the results, facilitating comparison across all studies. If the scales in different studies had varying directions (e.g., some scales were positively correlated with outcome measures while others were negatively correlated), the mean of the measures from negatively correlated scales was multiplied by –1 to ensure consistency (31).

Statistical heterogeneity was assessed using the chi-square test and the *I²* statistic. An *I²* value < 50% was considered indicative of low heterogeneity, whereas a value ≥ 50% suggested moderate to high heterogeneity. Consequently, a random-effects model was applied in the presence of moderate or high heterogeneity; otherwise, a fixed-effects model was utilized (32).

For meta-analytic outcomes with no fewer than 10 included studies, we planned to assess potential publication bias using funnel plots generated by Review Manager version 5.4 and Egger’s regression test performed in Stata 15 (33). The asymmetry of the funnel plots and the significance level of Egger’s test (*p* < 0.05) indicate the presence of publication bias. The trim and fill method (34) was applied if a publication bias was found. Funnel plots were constructed for each outcome category in this meta-analysis.

### 2.7 Risk of bias and sensitivity analysis

Two researchers independently evaluated the methodological quality of the studies. The Cochrane Bias Risk Assessment Tool (RoB 1.0) was used to evaluate random sequence generation, allocation concealment, investigator and subject blinding, outcome blinding, data integrity, selective reporting, and other biases (27). To ensure the robustness of the meta-analysis results, a sensitivity analysis was performed by excluding studies with high heterogeneity. This analysis employed a one-by-one exclusion method to assess the impact of individual studies on the overall results, aiming to identify sources of high heterogeneity and verify the reliability of the findings.

## 3 Results

### 3.1 Study selection

The PRISMA flowchart describing the selection process, including reasons for exclusion, is presented in **Figure 1**. The literature search was conducted in PubMed, Embase, Cochrane Library, Scopus, and Web of Science, yielding a total of 1,182 references. After removing duplicates, 415 articles remained. Following screening of titles and abstracts, 383 articles were excluded, leaving 32 articles. Upon full-text review, 18 articles were excluded due to lack of ADHD diagnosis (n = 4), being systematic reviews (n = 2), lack of RCTs (n = 10), or being protocol studies (n = 2). Ultimately, 14 articles were included in the meta-analysis. The risk of bias was assessed using the Cochrane Collaboration tool for assessing the risk of bias embedded in Review Manager version 5.4 (**Figures 2A, B**).

**Figure 1:**
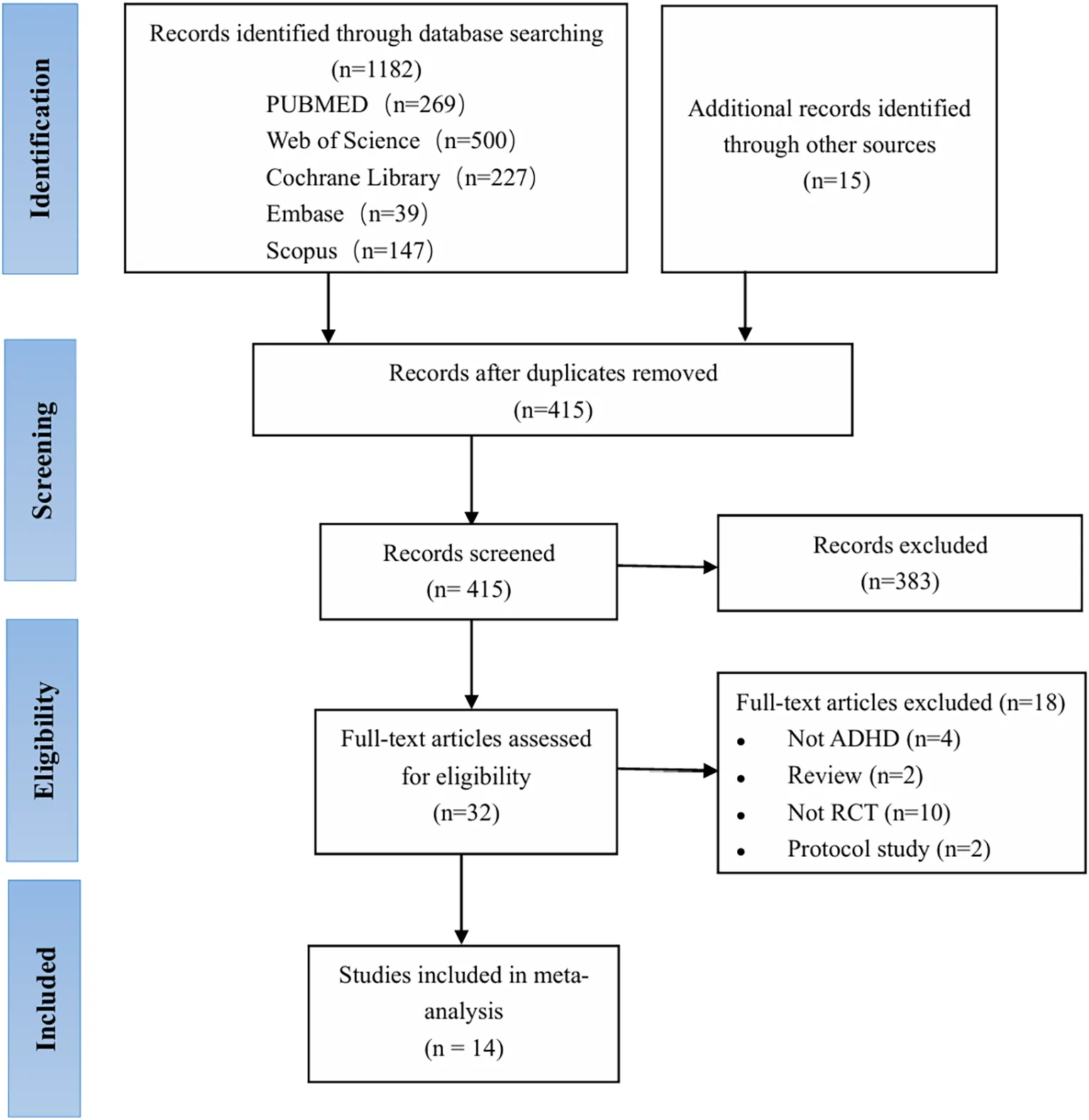
PRISMA flowchart reporting the screening process. RCT, randomized controlled trial.

**Figure 2:**
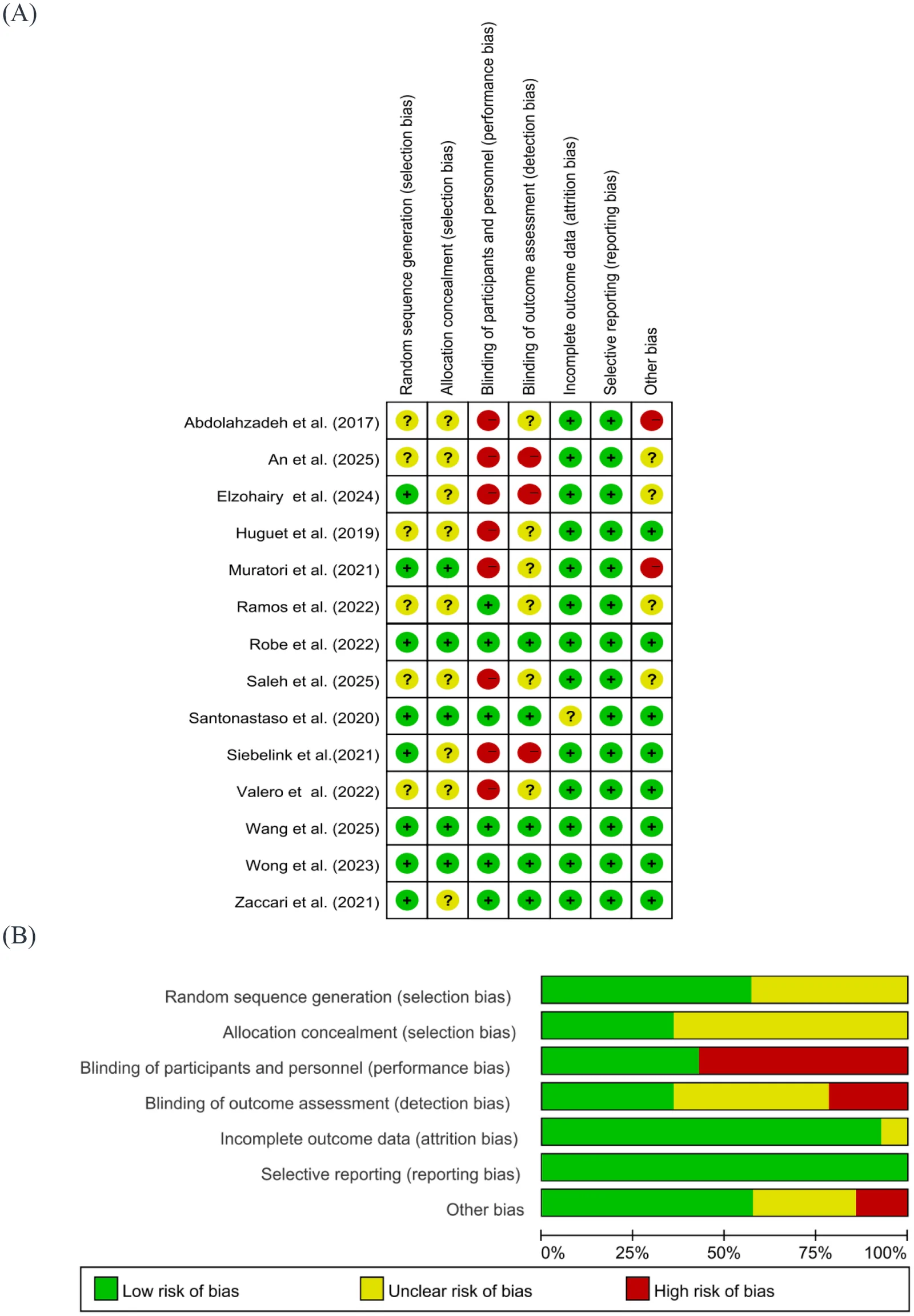
**(A)** Risk of bias summary. **(B)** Risk of bias graph. Risk of bias summary and graph, highlighting that while most included studies utilized proper random sequence generation, blinding of participants and personnel remained a major methodological limitation across the included trials.

### 3.2 Literature search and study characteristics

The included studies covered 8 countries, including Iran (35, 36), Spain (37, 38), Italy (39–41), the Netherlands (42), the United States (43), Romania (44), China (19, 45, 46), and Egypt (47). These studies were published between 2017 and 2025, with sample sizes ranging from 25 to 138. The overall age range of participants across the studies was 6 to 18 years.

The majority of studies recruited a significantly higher proportion of males than females with ADHD (19, 37–39, 42–46), while only one study recruited a significantly higher proportion of females than males with ADHD (47). Furthermore, one study recruited exclusively female adolescents with ADHD (35), two studies recruited exclusively male children with ADHD (36, 40), and one study did not describe the gender of the participants (41).

The majority of mindfulness interventions were based on the MBSR program and MBCT (19, 35, 37, 39–41, 44, 46). Three studies adopted MYmind, a standardized family mindfulness intervention program specifically designed to address core issues in children with ADHD and their parents (38, 42, 45). Additionally, one study utilized MindUP, a manualized mindfulness and social-emotional learning program (43). The duration of individual sessions ranged from 10 to 90 minutes, with most sessions lasting 90 minutes (19, 35, 36, 40, 42, 45). Intervention frequency ranged from one to three times per week, with the majority of studies conducting sessions once a week (19, 35–38, 40, 42, 44, 45, 47). The total duration of the interventions ranged from 1 to 12 weeks, with most interventions lasting 8 weeks (35–41, 45, 46) (see **Supporting Information Table S1**).

A synthesis of the included studies reveals both emerging patterns and significant methodological heterogeneity. Regarding similarities, the majority of interventions were structured around 8-week protocols (e.g., MBSR and MBCT), suggesting a general consensus on the optimal dosage and format for delivering MBIs to children and adolescents with ADHD. However, pronounced differences emerged in intervention components and control group designs. While some studies evaluated standalone mindfulness training against a waitlist, others integrated MBIs with standard care or behavioral therapies. Furthermore, the control conditions varied—from passive waitlists to active controls like cognitive behavioral therapy (CBT) These discrepancies likely contributed to the substantial statistical heterogeneity observed in our pooled analyses.

### 3.3 Effects on ADHD symptoms

This study pooled 8 effect sizes from 6 studies to assess ADHD symptoms using the Swanson, Nolan, and Pelham, Version IV (SNAP-IV), Conners’ Parent Rating Scale-Revised: Long Version (CPRS-R:L), Strengths and Weaknesses of ADHD Symptoms and Normal Behavior (SWAN), Child Behavior Checklist (CBCL), and Vanderbilt ADHD Diagnostic Parent Rating Scale (VADPRS) scales (19, 35, 39, 41, 45, 47). A random-effects meta-analysis revealed a significant reduction in ADHD symptoms in the mindfulness group (SMD = -1.30, 95% confidence interval (95% CI) [-2.14, -0.45], *p* = 0.003), but indicated high heterogeneity across studies (*I²* = 91%, *p* < 0.00001) (**Figure 3A**). From a clinical perspective, the magnitude of the pooled effect sizes provides valuable insights into the practical implications of MBIs. The large effect size observed for core ADHD symptoms (SMD = -1.30) suggests that mindfulness training may offer clinically meaningful reductions in symptom severity. However, this must be interpreted cautiously given the high heterogeneity (*I²* = 91%). The substantial heterogeneity observed for core ADHD symptoms (*I²* = 91%) warrants further exploration to provide more pragmatic clinical guidance. Upon stratifying the included studies into a children subgroup (mean age ≤ 12 years) and an adolescent subgroup (mean age > 12 years) based on the participants’ mean ages, we observed that the majority of studies in the children subgroup demonstrated a trend toward core symptom improvement. Meanwhile, the adolescent subgroup comprised only a single study, which nonetheless indicated that mindfulness interventions could ameliorate core symptoms. These observations suggest that MBIs may be particularly effective in younger pediatric populations, although the limited number of studies within each subgroup precludes definitive conclusions. Regarding intervention type, upon comparing standalone mindfulness interventions (e.g., MBSR or MBCT against treatment as usual) with adjunctive interventions (e.g., MYmind or MindUP), we found that the majority of the included studies evaluated standalone mindfulness interventions, which demonstrated positive outcomes in reducing core symptoms. Conversely, the adjunctive intervention subgroup comprised only a single study utilizing MYmind, which did not show a positive effect of mindfulness interventions on core symptoms. This discrepancy may partly account for the high heterogeneity observed in the pooled analysis.

**Figure 3:**
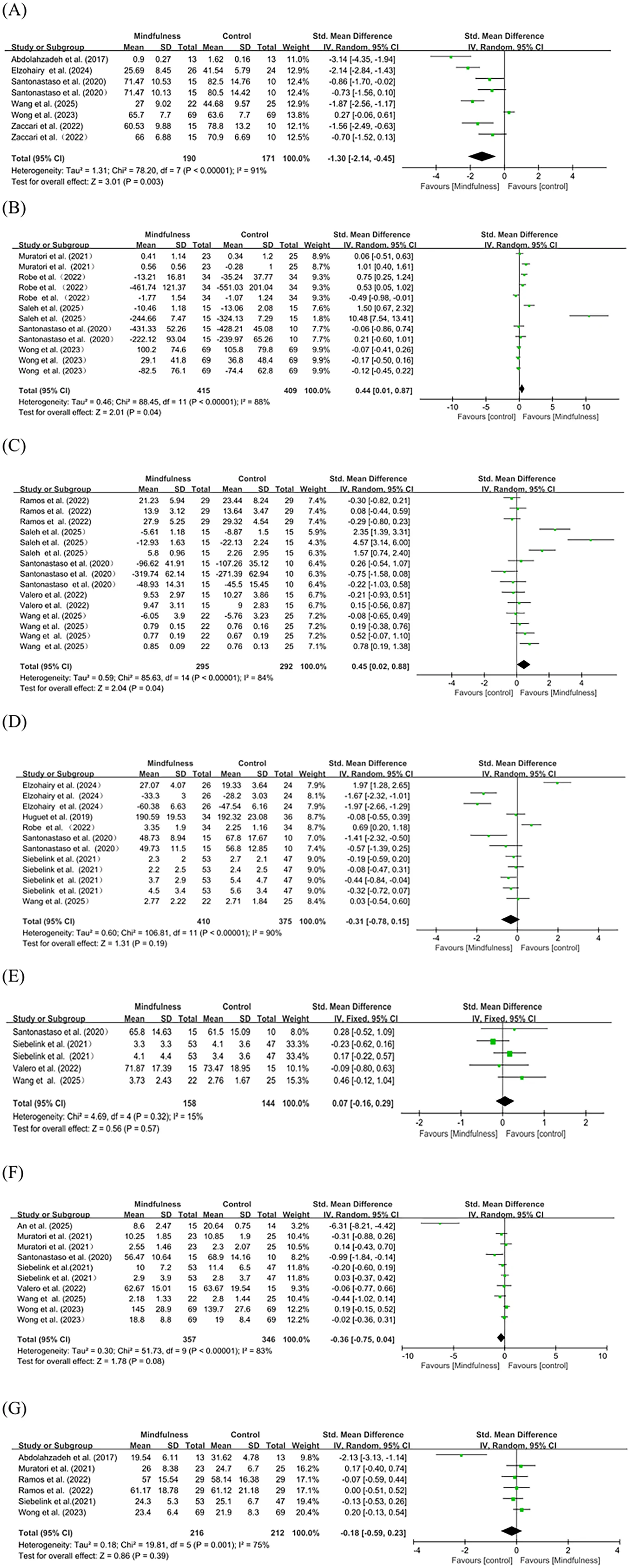
**(A)** Forest plots of effect size for ADHD symptoms. **(B)** Forest plots of effect size for attention network functions. **(C)** Forest plots of effect size for executive function. **(D)** Forest plots of effect size for emotional problems. **(E)** Forest plots of effect size for social problems. **(F)** Forest plots of effect size for oppositional defiant, disruptive, and aggressive behaviors. **(G)** Forest plots of effect size for mindfulness skills. Forest plots of effect sizes demonstrating that MBIs significantly improved core ADHD symptoms, attention network functions, and executive function, while showing no significant effects on emotional problems, social problems, externalizing behaviors, or mindfulness skills.

### 3.4 Neurocognitive functions

#### 3.4.1 Effects on attention network functions

Based on 12 effect sizes from 5 studies, neuropsychological tests including the Continuous Performance Test-II (CPT-II), Bells Test, and Attention Network Test (ANT) were administered to assess attention network functions (36, 39, 40, 44, 45). The results showed that attention network functions in the mindfulness group were significantly higher than those in the control group (SMD = 0.44, 95% CI [0.01, 0.87], *p* = 0.04). Furthermore, heterogeneity testing indicated significant heterogeneity (*I²* = 88%, *p* < 0.00001) (**Figure 3B**), reflecting differences in outcome measures across the studies. Visual assessment of the funnel plot showed asymmetry (**Supplementary Figure S1A**), and Egger’s test was statistically significant (*p* = 0.04), suggesting potential publication bias. However, trim and fill analysis based on the random-effects model showed that the statistical significance of the pooled effect size remained unchanged (*p* = 0.039), indicating that publication bias did not affect the overall conclusion of the study (**Supplementary Figure S1B**).

#### 3.4.2 Effects on executive function

This study included 15 effect sizes from 5 studies. Outcomes related to executive function were assessed, including inhibitory control, working memory, and cognitive flexibility. Inhibitory control was evaluated using the Stroop, Stop, NEPSY-II, NIH Cognitive Toolbox Flanker, Go/No-Go, and Simon tasks (19, 38, 39, 43). Working memory was assessed with the N-back task, the NIH Cognitive Toolbox List Sorting Working Memory Test, and the Letter-Number Sequencing subtest of the WISC-IV (19, 38, 39, 43). Cognitive flexibility was measured using the NIH Cognitive Toolbox Dimensional Change Card Sort, Global-Local Set Shifting (GLSS) task, and the Wisconsin Card Sorting Test (WCST) (19, 36, 43).

The results showed that MBI had a significant positive effect on executive function (SMD = 0.45, 95% CI [0.02, 0.88], *p* = 0.04). Significant heterogeneity was detected (*I²* = 84%, *p* < 0.00001) (**Figure 3C**). Visual inspection of the funnel plot suggested possible publication bias (**Supplementary Figure S2A**), and Egger’s test reached statistical significance (*p* = 0.008), further supporting the presence of potential publication bias. However, trim-and-fill analysis based on the random-effects model indicated that the statistical significance of the pooled effect size remained unchanged (*p* = 0.039), suggesting that publication bias did not alter the overall conclusion of the study (**Supplementary Figure S2B**).

For neurocognitive functions, the moderate effect sizes for attention network function (SMD = 0.44) and executive function (SMD = 0.45) indicate a tangible, though less pronounced, benefit. While these cognitive improvements are statistically significant, whether they reach the threshold for clinically significant changes in daily life remains uncertain.

### 3.5 Effects on emotional problems

A total of 12 effect sizes from 6 studies were included. Emotional problems such as emotion regulation, anxiety, and shyness were assessed using tools including the CBCL, CPRS-R:L, Conners Parent Rating Scale (CPRS), Conners Teacher Rating Scale (CTRS), Visual Analogue Scale (VAS), Emotion Regulation Checklist (ERC), and Strengths and Difficulties Questionnaire (SDQ) (19, 37, 39, 42, 44, 47). The results showed that although the overall effect size favored mindfulness-based intervention (SMD = -0.31, 95% CI [-0.78, 0.15]), the difference did not reach statistical significance (*p* = 0.19). Significant heterogeneity was observed across studies (*I²* = 90%, *p* < 0.00001) (**Figure 3D**). Quantitative assessment via Egger’s linear regression test (*p* = 0.509), supplemented by visual inspection of funnel plot symmetry (**Supplementary Figure S3**), did not indicate significant publication bias.

### 3.6 Effects on social problems

Four studies used scales such as the CPRS-R:L, CPRS, CTRS, Conners 3rd Edition, and SDQ to assess social problems (19, 38, 39, 42). The pooled effect size showed a slight direction favoring the control group, but the difference in social problems between the mindfulness and control groups was not statistically significant (SMD = 0.07, 95% CI [-0.16, 0.29], *p* = 0.57) (**Figure 3E**).

### 3.7 Effects on oppositional defiant, disruptive, and aggressive behaviors

Ten effect sizes pertained to outcomes related to externalizing behavior problems (including oppositional defiance, aggression, and disruptive behavior) (19, 38–40, 42, 45, 46). MBIs had a positive impact on externalizing behavior, but there was no statistically significant difference between the mindfulness and control groups (SMD = -0.36, 95% CI [-0.75, 0.04], *p* = 0.08). Significant heterogeneity was detected (*I²* = 83%, *p* < 0.00001) (**Figure 3F**). Visual assessment of the funnel plot suggested potential publication bias (**Supplementary Figure S4A**), and Egger’s test was statistically significant (*p* = 0.004), further confirming the existence of potential publication bias. Trim and fill analysis based on the random-effects model showed a change in the statistical significance of the pooled effect size (from non-significant to significant, *p* = 0.002), indicating that the results of this study may be influenced by publication bias (**Supplementary Figure S4B**).

### 3.8 Mindfulness skills

Six comparisons from 5 studies measured mindfulness outcomes using the Mindful Attention Awareness Scale (MAAS) and the Child and Adolescent Mindfulness Measure (CAMM) (35, 40, 42, 43, 45). The pooled effect size favored the control group but did not reach statistical significance (SMD = -0.18, 95% CI [-0.59, 0.23], *p* = 0.39). Significant heterogeneity was detected (*I²* = 75%, *p* = 0.001) (**Figure 3G**).

The small, non-significant effect sizes for emotional problems (SMD = -0.31), externalizing behaviors (SMD = -0.36), and mindfulness skills (SMD = -0.18) suggest that, at present, MBIs may not be sufficiently potent as standalone treatments for these specific domains in pediatric ADHD. Practically, these findings imply that while MBIs hold promise as an adjunctive intervention for core symptoms and specific cognitive deficits, they should not replace standard multimodal treatments targeting emotional and behavioral comorbidities. Clinicians should consider MBIs as a complementary approach.

A critical emerging pattern from our synthesis is the discrepancy between caregiver-reported core symptoms and objective neurocognitive assessments. Although significant improvements were observed in parent/teacher-rated ADHD symptoms, the positive effects on objective attention and executive function tasks, despite being statistically significant, exhibited high heterogeneity. This suggests that while MBIs may improve core symptoms, translating these improvements directly into underlying neurocognitive mechanisms requires more standardized objective evaluations. Additionally, the lack of significant effects on secondary outcomes (e.g., emotional and behavioral problems) may be attributed to the variability in assessment scales and the predominance of short-term follow-ups, as improvements in these functional domains typically require sustained practice to yield enduring benefits. These patterns underscore the necessity for future RCTs to adopt standardized MBI protocols and long-term objective evaluations to better determine the specific efficacy of mindfulness.

### 3.9 Sensitivity analysis

We conducted a one-by-one sensitivity analysis to evaluate the impact of each study on the SMD regarding ADHD symptoms (**Figure 4A**), attention (**Figure 4B**), executive function (**Figure 4C**), emotional problems (**Figure 4D**), oppositional defiant, disruptive, and aggressive behaviors (**Figure 4E**), and mindfulness skills (**Figure 4F**). The sensitivity analysis results indicated that the statistical significance of the SMDs for ADHD symptoms, attention, executive function, emotional problems, oppositional defiant, disruptive, and aggressive behaviors, and mindfulness skills remained unchanged after the removal of any single study. However, heterogeneity for oppositional defiant, disruptive, and aggressive behaviors was substantially reduced after excluding data reported by An et al. in 2025 (*I²* = 22%, *p* = 0.37) (46). Furthermore, heterogeneity for mindfulness skills disappeared (*I²* = 0%, *p* = 0.64) after excluding data reported by Abdolahzadeh et al. (35), suggesting that the aforementioned studies were the primary sources of heterogeneity.

**Figure 4:**
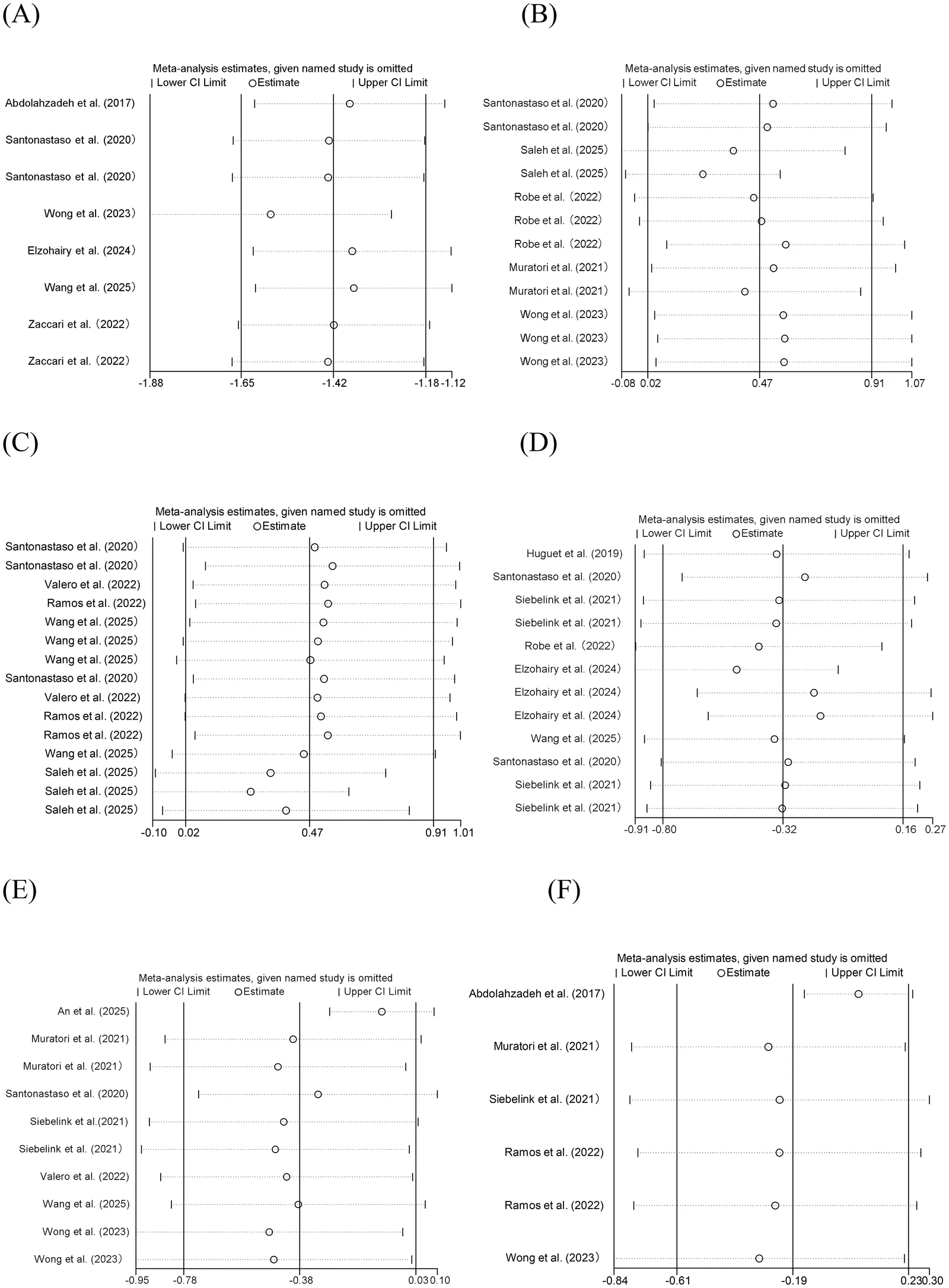
Sensitivity analysis. **(A)** ADHD symptoms, **(B)** attention, **(C)** executive function, **(D)** emotional problems, **(E)** oppositional defiant, disruptive, and aggressive behaviors, and **(F)** mindfulness skills. Sensitivity analysis confirming the robustness of the overall effect sizes for all outcomes, indicating that the statistical significance remained unchanged after the removal of any single study, and identifying An et al. (46) and Abdolahzadeh et al. (35) as the primary sources of high heterogeneity for externalizing behaviors and mindfulness skills, respectively.

## 4 Discussion

We evaluated the efficacy of MBIs on core symptoms, ADHD-related comorbidities, and neurocognitive functions in children and adolescents with ADHD, based on 14 studies. To our knowledge, this is a comprehensive systematic review and meta-analysis of RCTs in this field to date. Due to the increasing volume of relevant publications in recent years, this study was able to include more RCTs compared to previous systematic reviews and meta-analyses. Our findings extend those of a 2022 meta-analysis (28).

Consistent with the results of previous systematic reviews and meta-analyses (23, 24, 27–29, 48–50). ADHD symptoms remain the most extensively investigated outcome. Our analysis aggregated six MBI studies and suggested a potential moderate-to-large effect of mindfulness interventions on ADHD symptoms in children and adolescents, aligning with prior literature (23, 24, 28, 48–50).

In addition to ADHD symptoms, several studies have incorporated neurocognitive function as an outcome measure. Results from five studies included in this meta-analysis suggest that MBIs may improve attentional network function, yielding a moderate effect size (36, 39, 40, 44, 45). Furthermore, five studies evaluated the efficacy of MBIs on executive function. Our findings indicate a potential positive signal of MBIs on executive function in children and adolescents with ADHD, including inhibitory control (19, 38, 39, 43), working memory (19, 38, 39, 43), and cognitive flexibility (19, 36, 43), with a moderate effect size. To our knowledge, this is one of the few meta-analyses of RCTS to investigate the impact of MBIs on improving executive function in children and adolescents with ADHD, providing an update to the prior systematic review focused on observational studies (51). Although our data suggest that children and adolescents randomized to the mindfulness group tended to show better outcomes than controls, the current evidence is insufficient to draw definitive conclusions regarding the efficacy of MBIs on core symptoms and neurocognitive functions. The apparent therapeutic benefit requires validation through more rigorously designed, large-scale trials with highly standardized protocols.

The improvements in attention and executive function observed in our analysis provide direct empirical support for the psychological mechanism of enhanced top-down cognitive control. However, the underlying neurobiological mechanisms—such as functional changes in the prefrontal cortex or default mode network, and dopaminergic regulation—were not directly measured by the included RCTs. Therefore, these biological mechanisms remain theoretical inferences drawn from previous studies. As stated in our synthesis, translating clinical improvements directly into underlying neurocognitive mechanisms requires more standardized objective evaluations in future trials.

This meta-analysis indicates that emotional health, social relationships, externalizing behaviors, and mindfulness skills did not show significant improvements, which is consistent with the findings of a previous meta-analysis (28). The non-significant findings may be attributed to: (1) the lack of long-term follow-up data, as these domains require sustained practice to show enduring benefits beyond the immediate intervention phase; and (2) high methodological variability. The use of non-uniform assessment scales hindered direct comparability, while significant heterogeneity in MBI types, dosages, and combinations with standard care increased inter-study variability. This methodological diversity likely obscured actual improvements, making it difficult for pooled effects to reach significance.

Using the GRADE framework, the overall certainty of evidence for our primary findings—specifically the effects of MBIs on ADHD symptoms, attention network functions, and executive function—was rated as low. This downgrading was primarily due to a serious risk of bias (e.g., lack of blinding in most included RCTs), substantial heterogeneity, and potential publication bias detected in several outcomes. Similarly, the certainty of evidence for secondary outcomes, including emotional, social, and behavioral problems, was assessed as low. Consequently, these GRADE ratings underscore our conclusion that while MBIs show potential benefits, the current evidence is insufficient to draw definitive conclusions, and further high-quality, standardized trials are required.

## 5 Limitations

The studies included in this meta-analysis exhibited certain methodological limitations. First, several studies did not report specific methods for random allocation or allocation concealment. Second, the majority of studies lacked blinding of researchers and participants, which may have resulted in biased outcome assessments. It is important to distinguish the impact of this limitation across outcome types. Subjective parent/teacher ratings (e.g., SNAP-IV, CPRS) are highly susceptible to performance and detection bias in the absence of blinding, as caregivers aware of the intervention assignment may report greater improvements due to expectancy effects. Conversely, objective neurocognitive tasks (e.g., CPT-II, Stroop), while subject to other sources of variability, are generally less influenced by the rater’s awareness of treatment allocation compared to behavioral questionnaires. Third, potential publication bias regarding the effects of MBIs on externalizing behaviors in children and adolescents with ADHD was detected, raising concerns regarding the impact of MBIs in this population. Finally, a potential limitation of this study is the failure to include grey literature by conducting a dedicated search in clinical trial registries (e.g., ClinicalTrials.gov or World Health Organization International Clinical Trials Registry Platform). This may introduce publication bias, as relevant studies with non-significant findings might have been missed. Future reviews could benefit from incorporating these sources to provide a more comprehensive overview of the evidence.

## 6 Conclusion

In conclusion, this systematic review and meta-analysis suggests that MBIs may offer potential benefits for core ADHD symptoms and specific neurocognitive functions (attention and executive functions) in children and adolescents. However, these positive findings must be counterbalanced by remaining uncertainties: the effects on emotional health, social relationships, externalizing behaviors, and mindfulness skills remain inconclusive, and the primary findings are limited by substantial heterogeneity, a serious risk of bias, and low certainty of evidence. Therefore, while MBIs hold promise as a complementary component within multimodal ADHD management, the current evidence is insufficient to draw definitive conclusions. Future research must employ standardized protocols, rigorous methodological designs, and long-term follow-ups to address these uncertainties and verify the true efficacy of MBIs in this population. Regarding the specific format of future protocols, it is crucial to distinguish between family-based interventions (e.g., MYmind) and individual models (e.g., MindUP). While family-based models actively involve parents and address the dyadic nature of ADHD management—which may facilitate the generalization of skills to the home environment—they are often more resource-intensive. Conversely, individual models offer superior scalability within educational settings. Given the substantial heterogeneity observed in our current analysis, which included a mix of these delivery formats, the data are currently insufficient to definitively prioritize one model over the other. Therefore, future research should not only standardize protocols but also prioritize head-to-head comparative trials to determine if the theoretical benefits of family engagement translate into significantly superior clinical outcomes compared to standardized school-based curricula.

## Data Availability

The original contributions presented in the study are included in the article/**Supplementary Material**. Further inquiries can be directed to the corresponding author.
