# Supplementary material for: Efficacy of mindfulness-based interventions for children and adolescents with attention deficit hyperactivity disorder: a systematic review and meta-analysis
Source: Front Psychiatry. 2026 Sep 16;17:1894822. doi: 10.3389/fpsyt.2026.1894822 (PMC13623847; doi:10.3389/fpsyt.2026.1894822)
Supplement: Supplementary file 1 [file SupplementaryFile1.docx]

**Supporting information**

**Table S1.** Main characteristics of the included studies in the systematic review and meta-analysis.

**S1**. Search strategies

**Figure. S1A**. Funnel plot for attention network functions.

**Figure. S1B**. Funnel plot after trim and fill analysis for attention network functions.

**Figure. S2A.** Funnel plot for executive function.

**Figure. S2B**. Funnel plot after trim and fill analysis for executive function**.**

**Figure. S3.** Funnel plot for emotional problems.

**Figure. S4A**. Funnel plot for oppositional defiant, disruptive, and aggressive behaviors.

**Figure. S4B.** Funnel plot after trim and fill analysis for oppositional defiant, disruptive, and aggressive behaviors.

**Table S1.** Main characteristics of the included studies in the systematic review and meta-analysis.

**S1. Search strategies**

**Population**: (“Children” OR “Child” OR “Adolescent” OR “Adolescents” OR “Youth” OR “Youths” OR “Teens” OR “Teen” OR “Teenagers” OR “Teenager”) AND ( “Attention Deficit Disorder” OR “ADHD” OR “ADDH” OR “Attention Deficit Disorders with Hyperactivity” OR “Attention Deficit Hyperactivity Disorders” OR “Attention Deficit Hyperactivity Disorder” OR “Attention Deficit-Hyperactivity Disorder” OR “Attention Deficit-Hyperactivity Disorders” OR “Deficit-Hyperactivity Disorder, Attention” OR “Deficit-Hyperactivity Disorders, Attention” OR “Disorder, Attention Deficit-Hyperactivity” OR “Disorders, Attention Deficit-Hyperactivity” OR “Hyperkinetic Syndrome” OR “Syndromes, Hyperkinetic” OR “Attention Deficit Disorders” OR “Deficit Disorder, Attention” OR “Deficit Disorders, Attention” OR “Disorder, Attention Deficit” OR “Disorders, Attention Deficit”)

**Exposure**: ("Mindfulness" OR “Meditation” OR “Mindfulness Practice” OR “Mindfulness Meditation” OR “Meditation, Mindfulness” OR “Mindfulness Meditations” OR “Mindfulness-Based Cognitive Therapy” OR “Cognitive Therapy, Mindfulness-Based” OR “Mindfulness-Based Cognitive Therapies” OR “Mindfulness Based Cognitive Therapy” OR “Therapy, Mindfulness-Based Cognitive” OR “MBCT” OR “Mindfulness-Based Stress Reduction” OR “Mindfulness Based Stress Reduction” OR “Mindfulness-Based Stress Reductions” OR “Stress Reduction, Mindfulness-Based” OR “MBSR Therapy” OR “MBSR Therapies” OR “Therapy, MBSR” OR “Mindfulness-Based Stress Reduction Therapy” OR “Mindfulness Based Stress Reduction Therapy” OR “MBSR” OR “Mindfulness-Based Intervention” OR “Mindfulness-Based Therapy” OR “MBT” OR “Mindfulness-Based Interventions” OR “MBIs” OR “MBI” OR “Family-Based Mindfulness Intervention” OR “Mindful Movement Intervention” OR “Mindful Walking and Eating” OR “Body Scan”).

(A)	(B)

**Figure S1.**

(A) Funnel plot for attention network functions. (B) Funnel plot after trim and fill analysis for attention network functions.

(A)	(B)

**Figure S2.**

(A) Funnel plot for executive function. (B) Funnel plot after trim and fill analysis for executive function**.**

**Figure S3.** Funnel plot for emotional problems.

(A)	(B)

**Figure S4.** (A) Funnel plot for oppositional defiant, disruptive, and aggressive behaviors. (B) Funnel plot after trim and fill analysis for oppositional defiant, disruptive, and aggressive behaviors.
